# Supplementary material for: Peripheral Blood Mononuclear Cell Populations Correlate with Outcome in Patients with Metastatic Breast Cancer
Source: Cells. 2022 May 13;11(10):1639. doi: 10.3390/cells11101639 (PMC9139201; doi:10.3390/cells11101639)
Supplement: Supplementary file 1 [file cells-11-01639-s001.zip › Larsson-et-al_cells-1671937_Supplemental-info_FINAL/Larsson-et-al_Supplemental-Info.pdf]

Peripheral blood mononuclear cell populations correlate with outcome in patients with metastatic breast cancer

Anna-Maria Larsson [1], Olle Nordström [2], Alexandra Johansson [1], Lisa Rydén [1,3], Karin Leandersson [4] and Caroline Bergenfelz [2,*]

1. Division of Oncology, Department of Clinical Sciences Lund, Lund University, SE-223 81 Lund, Sweden; anna-maria.larsson@med.lu.se (AML); al0531jo-s@student.lu.se (AJ); lisa.ryden@med.lu.se (LR)
2. Experimental Infection Medicine, Department of Translational Medicine, Lund University, SE-214 28 Malmö, Sweden; ol7840no-s@student.lu.se (ON); caroline.bergenfelz@med.lu.se (CB)
3. Department of Surgery, Skåne University Hospital, SE-223 81 Lund, Sweden
4. Cancer Immunology, Department of Translational Medicine, Lund University, SE-214 28 Malmö, Sweden; karin.leandersson@med.lu.se (KL)

* Correspondence: caroline.bergenfelz@med.lu.se

SUPPLEMENTAL TABLES

Supplemental Table S1. Characteristics of the included patients. [a] De novo MBC denotes MBC at initial breast cancer diagnosis. [b] Distant recurrent MBC denotes MBC diagnosis after >0 years after the primary diagnosis. [c] Visceral metastasis is defined as involvement of lung, liver, peritoneal and/or pleura. [d] Progression is defined as progressive disease at three months evaluation, using modified RECIST 1.1 criteria. *Abbreviations:* ECOG; Eastern Cooperative Oncology Group, NHG; Nottingham Histological Grade, PT; primary tumor, ER; estrogen receptor, PR; progesterone-receptor, HER2; human epidermal growth factor receptor 2, MET; metastasis.

**Variable**	***Total N*=32**	**%**
Age		
< 65 years	17	53.1
≥65 years	15	46.9
Baseline ECOG		
0	22	68.8
1	6	18.8
2	4	12.5
Unknown	0	0.0
PT Tumor type		
Ductal	21	65.5
Lobular	6	18.8
Other	4	12.5
Unknown	1	3.1
PT NHG		
I	2	6.3
II	13	40.6
III	7	21.9
Unknown	10	31.3
PT ER status		
ER-negative	8	25.0
ER-positive	20	62.5
ER-unknown	4	12.5
PT PR status		
PR-negative	12	37.5
PR-positive	15	46.9
PR-unknown	5	15.6
PT HER2 status		
HER2-negative	16	50.0
HER2-positive	5	15.6
HER2-unknown	11	34.4
Type of MBC		
De novo [a]	5	15.6
Distant recurrent [b]	27	84.4
Metastatic sites, number		
< 3 metastatic sites	19	59.4
≥ 3 metastatic sites	13	40.6
Metastatic sites, localization		
Lymph nodes No / Yes	20 / 12	62.5 / 37.5
Lung No / Yes	20 / 12	62.5 / 37.5

Liver No / Yes	23 / 9	71.9 / 28.1
Bone No / Yes	7 / 25	21.9 / 78.1
Visceral [c] No / Yes	13 / 19	40.6 / 59.4
Bone only No / Yes	23 / 9	71.9 / 28.1
MET ER status		
ER-negative	1	3.1
ER-positive	27	84.4
ER-unknown	4	12.5
MET PR status		
PR-negative	16	50.0
PR-positive	11	34.4
PR-unknown	5	15.6
MET HER2 status		
HER2-negative	23	71.9
HER2-positive	3	9.4
HER2-unknown	6	18.8
Progression at 3 mo evaluation[d]		
Non-progression	25	78.1
Progression	3	9.4
Unknown	4	12.5

Supplemental Table S2. Antibodies used for flow cytometry with clone and dilution indicated. All from BD Biosciences unless otherwise stated. [a.] from Miltenyi Blood dendritic cell enumeration kit that also includes CD14 and CD19 to exclude monocytes and B lymphocytes, respectively.

**Antibody**	**Clone**	**Dilution**
CD14-FITC	M5E2	1:10
CD16-PE	3G8	1:20
HLA-DR-APC	G46-6	1:50
CD3-FITC	HIT3a	1:25
CD3-APC	HIT3a	1:20
CD4-PE	RPA-T4	1:25
CD8-PE	HIT8a	1:25
CD25-FITC	2A3	1:10
CD127-biotin / SA-APC	HIL-7R-M21	1:10 / 1:10
CD56-APC	B159	1:10
CD19-FITC	HIB19	1:20
CD33-APC	WM53	1:10
BDCA-1 (CD1c)[a]	NA[a]	1:10
BDCA-2 (CD303)[a]	NA[a]	1:10
BDCA-3 (CD141)[a]	NA[a]	1:10

Supplemental Table S3. Summary of the analyzed immune cell populations. Circulating immune cell populations were analyzed by flow cytometry (see Supplementary Figure 1 for gating strategies). Values represent percentage of immune cell population of PBMCs or, where indicated, of all $CD4^+$ T cells. [a.] Ratio calculated as percentage of $CD4^+$ cells of all lymphocytes / $CD8^+$ cells of all lymphocytes. [b.] Ratio calculated as percentage of $CD14^+CD16^+$ cells / $CD14^+CD16^-$ cells. Median values with SEM and number of patients analyzed shown.

**Immune cell population or ratio**	***N***	**Median ± SEM**
% All T lymphocytes ($CD3^+$ cells)	32	23.23 % ± 2.05
% Cytotoxic T lymphocytes ($CD8^+$ cells)	32	6.59 % ± 0.96
% T helper cells ($CD4^+$ cells)	32	14.63 % ± 1.31
% Tregs ($CD4^{++}CD25^+CD127^{low/-}$ cells of all $CD4^{++}$ cells)	31	3.68 % ±0.42
% $CD8^+$ cells of all $CD3^+$ T lymphocytes	32	34.34 % ± 2.02
% $CD4^+$ cells of all $CD3^+$ T lymphocytes	32	64.54 % ± 2.66
Ratio $CD4^+$ T cells / $CD8^+$ T cells[a]	32	1.86 ±0.32
% B lymphocytes ($CD19^+$ cells)	26	4.34 % ± 0.86
% NK cells ($CD56^+CD3^-$ cells)	32	2.82 % ± 0.49
% NKT cells ($CD56^+CD3^+$ cells)	32	0.53 % ± 0.28
% Classical monocytes ($CD14^{++}CD16^-$ cells)	27	7.36 % ±0.91
% Intermediate monocytes ($CD14^{++}CD16^{+/++}$ cells)	27	2.59 % ± 0.46
% Non-classical monocytes ($CD14^+CD16^{++}$ cells)	27	0.27 % ± 0.06
Ratio $CD16^+$ / $CD16^-$ monocytes[b]	27	0.23 ± 0.05
% MDC1 (BDCA-1^+ cells)	31	0.31 % ± 0.05
% MDC2 (BDCA-3^+ cells)	31	0.02 % ± 0.01
% PDC (BDCA-2^+ cells)	31	0.58 % ±0.12

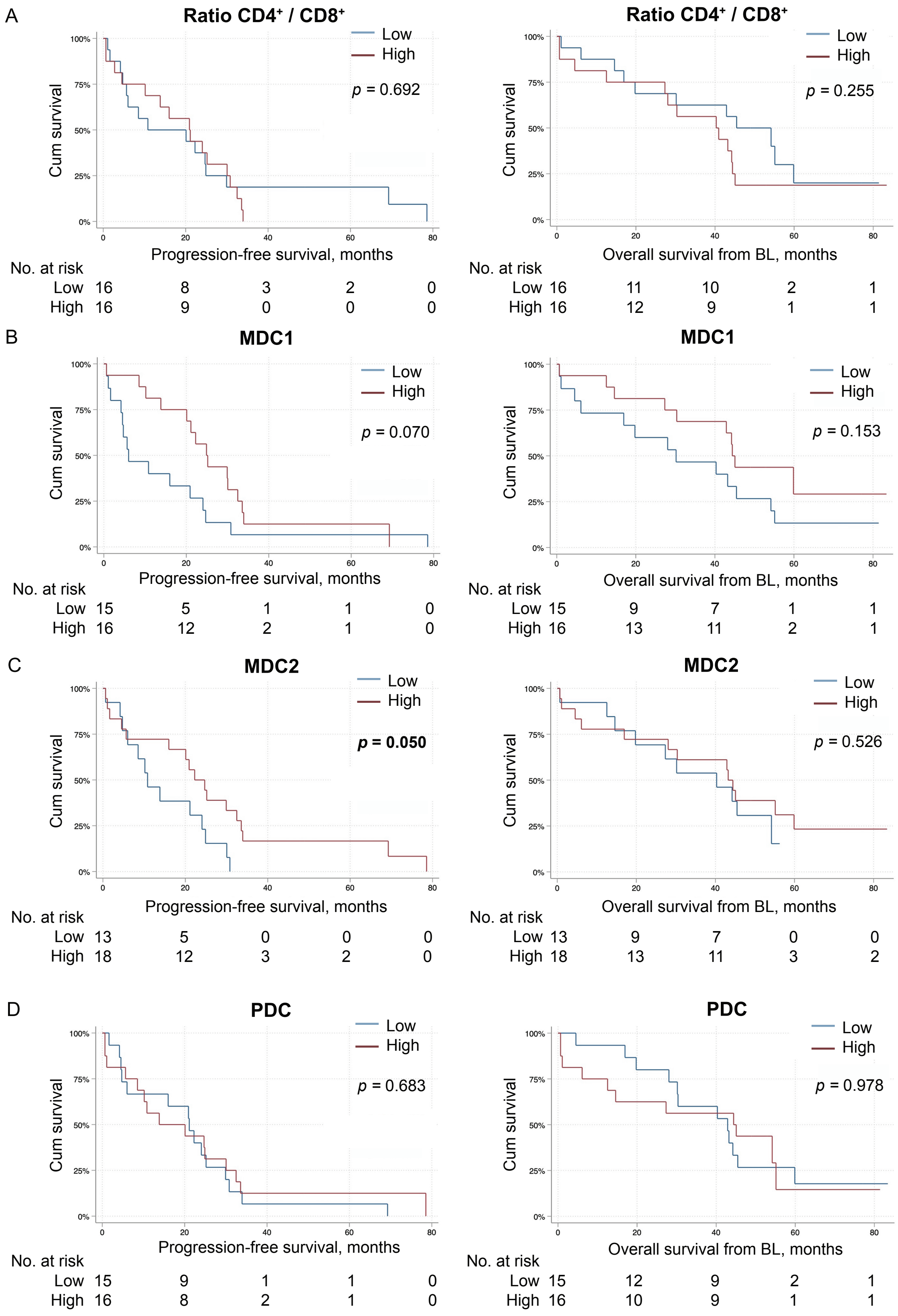

Supplemental Figure S1. High levels of systemic MDC1 associate with improved progression-free survival. Kaplan-Meier curves of progression-free survival (PFS; left panels) and overall survival (OS) from baseline (right panels) according to the levels (percentages) of indicated immune cell population in MBC patients. **A**, Ratio of % $CD4^{++}$ T-helper cells / % $CD8^{+}$ CTLs. **B**, BDCA-1^{+} MDC1. **C**, BDCA-3^{+} MDC2. **D**, BDCA-2^{+} PDC. *N*=31 for all populations except for $CD4^{+}/CD8^{+}$ ratio (*N*=32). Statistics by Log-rank test, *p*-val-ues < 0.05 highlighted in bold.

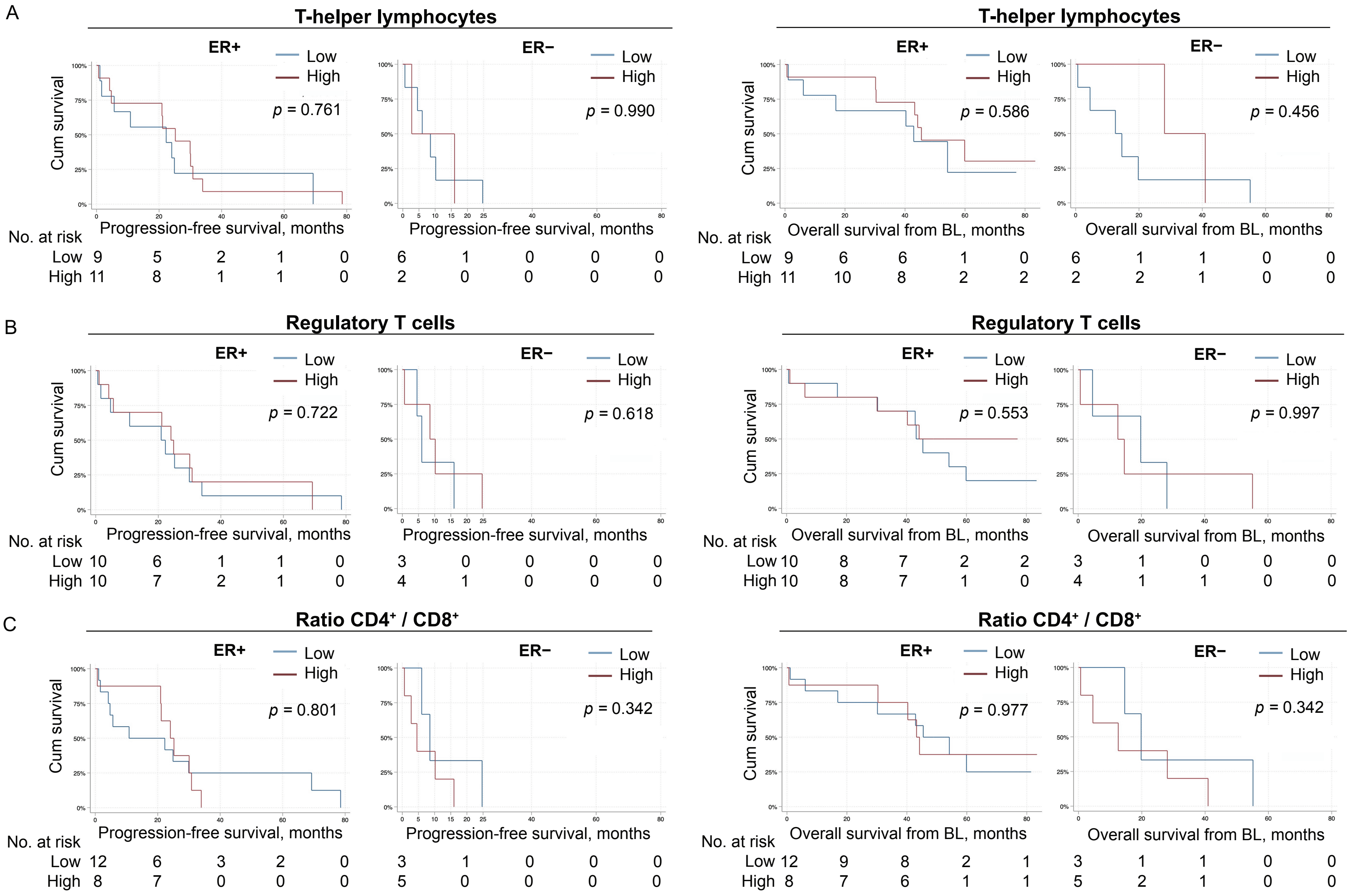

Supplemental Figure S2. The levels of T-helper or T_{regs} do not associate with survival in patients with ER-positive or ER-negative primary tumors. Kaplan-Meier curves of progression-free survival (PFS; left panels) and overall survival (OS, right panels) according to the levels of indicated immune cell populations in patients with MBC stratified for primary tumor ER status. **A**, $CD4^+$ T-helper cells. **B**, $CD4^+CD25^+CD127^{low/-}$ cells of $CD4^+$ cells. **C**, ratio of % $CD4^+$ T-helper cells / % $CD8^+$ CTLs. *N*=20 ER-positive and *N*=8 ER-negative for all populations except for T_{regs} (*N*=20 and *N*=7, respectively). Statistics by Log-rank test, *p*-values < 0.05 highlighted in bold.

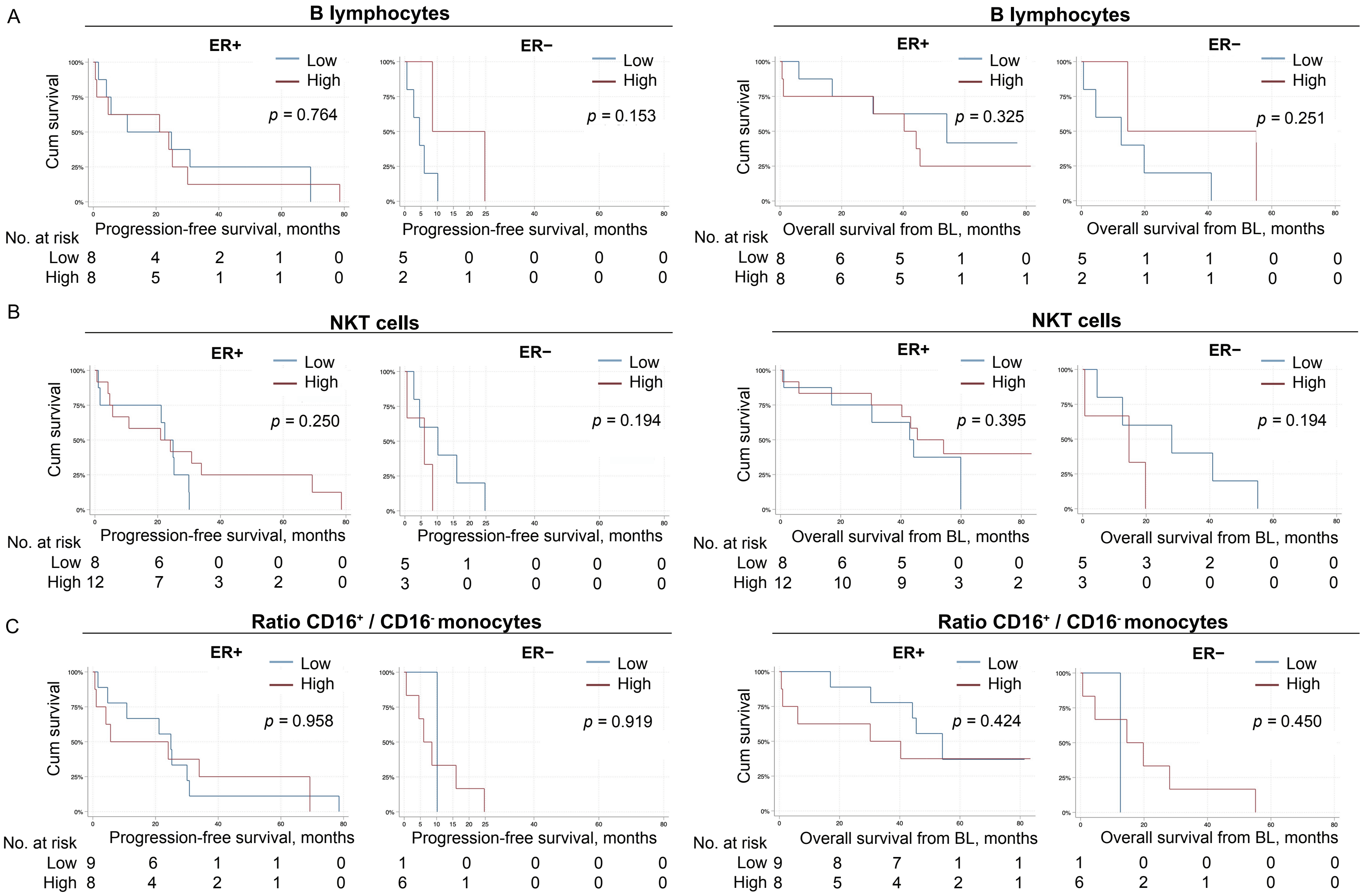

Supplemental Figure S3. The levels of B lymphocytes or NKT cells do not associate with survival in patients with ER-positive or ER-negative primary tumors. Kaplan Meier curves of progression-free survival (PFS; left panels) or overall survival (OS) from baseline (right panels) according to the levels of indicated immune cell populations in patients with MBC stratified for primary tumor ER status. **A**, $CD19^{+}$ B lymphocytes, *N*=16 ER-positive and *N*=7 ER-negative. **B**, $CD56^{+}CD3^{+}$ NKT cells, *N*=20 ER-positive and *N*=8 ER-negative. **C**, ratio of % $CD16^{+}$ monocytes / % $CD16^{-}$ monocytes, *N*=17 ER-positive and *N*=7 ER-negative. Statistics by Log-rank test, with *p*-values indicated.

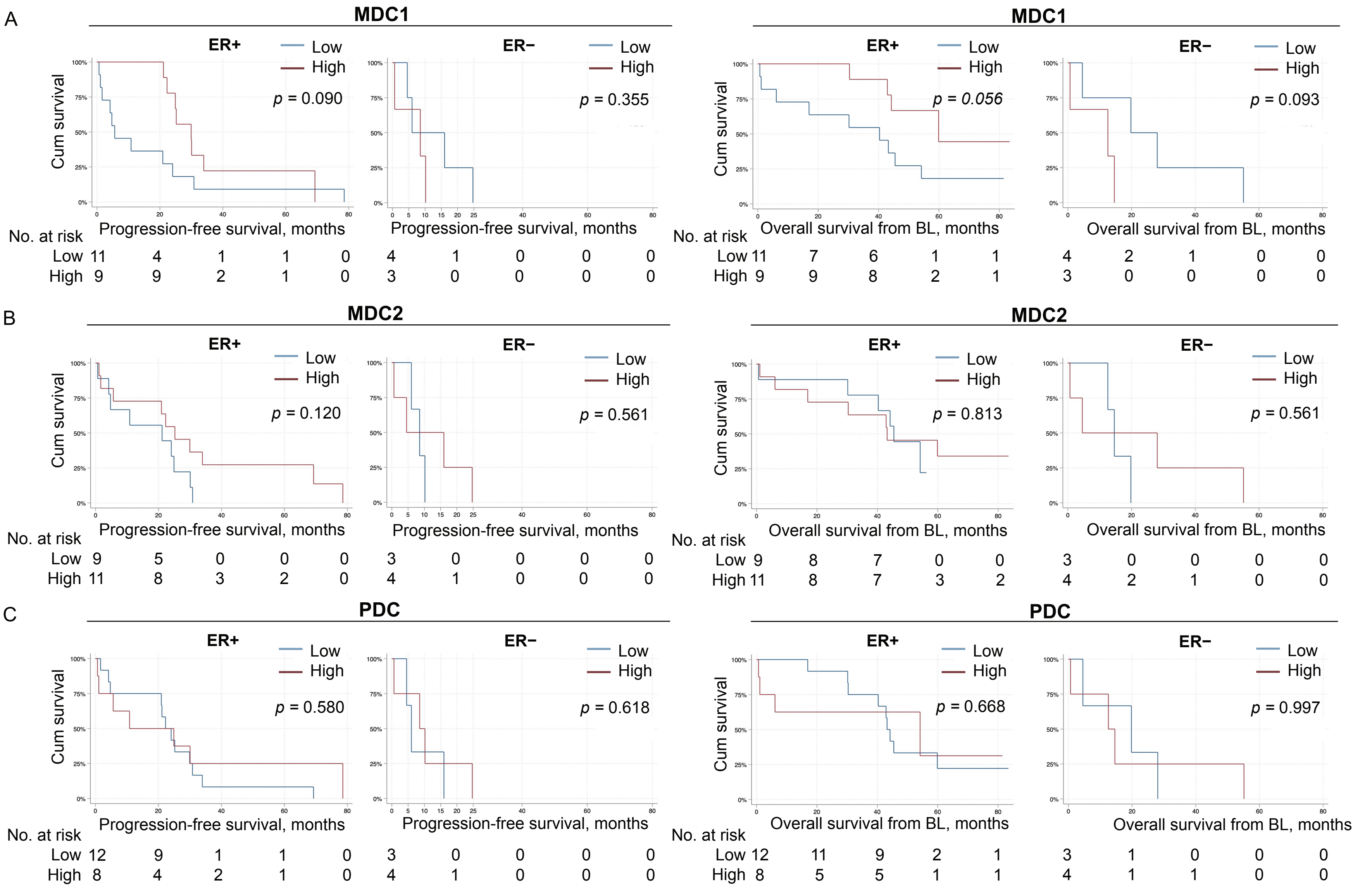

Supplemental Figure S4. The levels of MDC1 tend to associate with survival. Kaplan Meier curves of progression-free survival (PFS; left panels) or overall survival (OS) from baseline (right panels) according to the levels of indicated blood DC population in patients with MBC stratified for primary tumor ER status. **A**, BDCA-1^{+} MDC1. **B**, BDCA-3^{+} MDC2. **C**, BDCA-2^{+} PDC. N=20 ER-positive and N=7 ER-negative for all populations. Statistics by Log-rank test, with p-values indicated.
